# A Case Series of Round Block Techniques for Large, Recurrent, and Multicentric Benign Breast Diseases

**DOI:** 10.7759/cureus.60416

**Published:** 2024-05-16

**Authors:** Chitra R, Yuvaraj Karthick, Kamalraj Sundaramoorthy, Anupama Mohankumar

**Affiliations:** 1 Surgery, PSG (Peelamedu Soma Naidu Govidasamy Naidu) Institute of Medical Sciences and Research, Coimbatore, IND; 2 General Surgery, Trichy SRM (Sri Ramasamy Memorial) Medical College Hospital and Research Centre, Trichy, IND; 3 Surgery, PSG (Peelamedu Soma Naidu Govidasamy Naidu) Institute of Medical Science and Research, Coimbatore, IND; 4 School of Medicine, PSG (Peelamedu Soma Naidu Govidasamy Naidu) Institute of Medical Sciences and Research, Coimbatore, IND

**Keywords:** recurrent phyllodes, phyllodes, giant fibroadenoma, multicentric fibroadenoma, benign breast lesions

## Abstract

Benign breast diseases are a common presentation in the breast clinic outpatient department. These diseases, including giant fibroadenoma, multiple fibroadenoma in different quadrants, and large or recurrent phyllodes tumors, pose challenges in surgical management. We present a case series of 16 patients aged 19 to 63 years (average age, 41.5 years) who presented with breast lumps and underwent surgery using the round block technique for benign breast diseases at our institute between November 2019 and March 2024. Prior to surgery, all patients had clinical, radiological, and pathological assessments. Age, duration of lump, and detailed menstrual, obstetric, and family history of each patient were recorded. Eight (50%) of the patients had phyllodes tumor, four (31.25%) had fibroadenoma, three (18.75%) had both fibroadenoma and phyllodes tumor, and one (6.25%) had adenolipoma. The average size of tumors was 7.5 cm in our study. During postsurgical follow-up, none of the patients had nipple areola necrosis, and they reported that nipple sensation was acceptable. A mastectomy was avoided in all circumstances. Good cosmetic outcomes and clear margin status are achievable using the round block technique.

## Introduction

A breast lump may indicate benign or malignant disease, but in more than 90% of cases presenting in breast clinics, the cause is benign [1]. The most common benign breast diseases that present with a breast lump include fibroadenoma, phyllodes tumor, cysts, intraductal papilloma, lipoma, and abscess. Giant fibroadenoma, multiple fibroadenomas in different quadrants, and large or recurrent phyllode tumors are challenges in surgical management. Such lesions are difficult to excise by typical surgical techniques, which also have the likelihood of poor cosmetic outcomes.

Giant fibroadenomas that are >5 cm in size account for 0.5% to 2% of fibroadenomas and are most commonly found in adolescent patients [2]. Histopathologically, both fibroadenomas and phyllodes tumors have stromal and epithelial proliferation. However, fibroadenomas have low stromal cellularity with pericanalicular and intracanalicular growth patterns, whereas phyllodes tumors have leaf-like structures and heterogenous stroma [3]. Differentiating giant fibroadenomas from phyllodes tumors is important because fibroadenomas require simple excision, while phyllodes tumors need wide local excision with at least 1 cm margins [4]. Pseudoangiomatous stromal hyperplasia, myofibroblastoma, and myoid hamartoma are less common in the differential diagnosis of fibroadenomas [5].

The round block technique, or donut lumpectomy, is an oncoplastic procedure. Along with radiotherapy and chemotherapy, oncoplastic breast surgery has been used since the 1980s for breast conservation surgery in the context of breast cancer to preserve the breast, achieve good oncological clearance, and obtain better aesthetic outcomes [6]. Oncoplastic breast surgeries include various plastic reconstructive methods after wide excision of breast lesions, as well as immediate reshaping of the breast. These surgeries allow for better cosmesis and complete resection of local disease [7].

The Krishna Clough classification of oncoplastic breast surgery divides it into two types of surgery [8]. Level 1, or displacement surgery, is defined by less than 20% of breast volume being excised, along with immediate reshaping of the breast. Level 2, or replacement surgery, involves the excision of more than 20% of the breast volume, followed by immediate reconstruction done by mammoplasty or by various local pedicle flaps.

Oncoplastic techniques can also be applied in cases of benign breast disease. The round block technique is a level 1 oncoplastic procedure that avoids long, visible scars along the Langer and Kraissl lines. Also known as the Benelli mastopexy, the technique was devised by Benelli [9] in 1990. It involves making a periareolar incision rather than radial or inframammary incisions and provides access to all quadrants of the breast. Thus, it enables the excising of large, recurrent, and multicentric benign breast lesions that are typically difficult to remove.

In this case series report, we used the round block technique, which has previously been shown to be useful in breast cancer surgery [10], to treat patients with benign breast disease. Our objectives in each case were to excise the lesion and retain breast symmetry and cosmetic appearance. Mastectomy was avoided, and outcomes were acceptable in all cases.

## Materials and methods

In our case series, 16 patients at our institute underwent the round block technique for benign breast disease between November 2019 and March 2024. Consent was obtained or waived by all participants in this study. The Institutional Human Ethics Committee of the PSG Institute of Medical Sciences and Research issued approval 22/269.

Prior to surgery, all patients underwent a triple assessment [11], which included a detailed clinical history and examination, radiological imaging, and histopathology. Demographic data, including name, outpatient number, inpatient number, age, duration of illness, menstrual history, family history, and obstetric history, were recorded. For patients under 40 years old, breast imaging was done using ultrasound, while mammograms were used for patients who were 40 years of age or older. Imaging yielded data on the Breast Imaging Reporting and Data System stage, the size of lesions, and the breast quadrant involved. Pathology examination was done by either fine-needle aspiration cytology or core needle biopsy (with or without ultrasound guidance).

The round block technique was done by the same surgeon in all cases. First, two concentric periareolar incisions were made around 1 cm apart. The skin between the incisions was de-epithelized, with only the epidermis being removed. The incision was deepened between the two concentric rings, but only near the lesions. In this way, the vascularity of the nipple-areolar complex was preserved. The lesions were removed by either excision (for fibroadenoma) or wide local excision (1-cm clearance for phyllodes tumors and recurrent lesions). The margins of the specimen were immediately marked with silk, and the specimen was sent for biopsy. If biopsy specimens revealed malignancy, the patient was excluded from the study. The remaining breast parenchyma was approximated, and wound approximation was done by purse string and subcuticular absorbable stitches.

Patients were discharged, on average, two to four days after surgery. Postoperative radiological imaging was done during the follow-up period, every three months for two years. The histopathology report, any complications, and the duration of follow-up were noted for all patients. Preoperative and postoperative images of both breasts were taken with the patient in a seated position. These photos were used to assess the cosmetic results. The patient’s faces were not included in any photos in order to mask their identity. Images or pictures were also obtained intraoperatively without revealing the identity of the patient.

## Results

Sixteen patients, aged 19 to 63 years, were operated on by the same surgeon in our institute, PSGIMS&R, Coimbatore. The average age of the patients was 41.5 years. None of the patients were found to have a malignant disease. 

**Figure 1:**
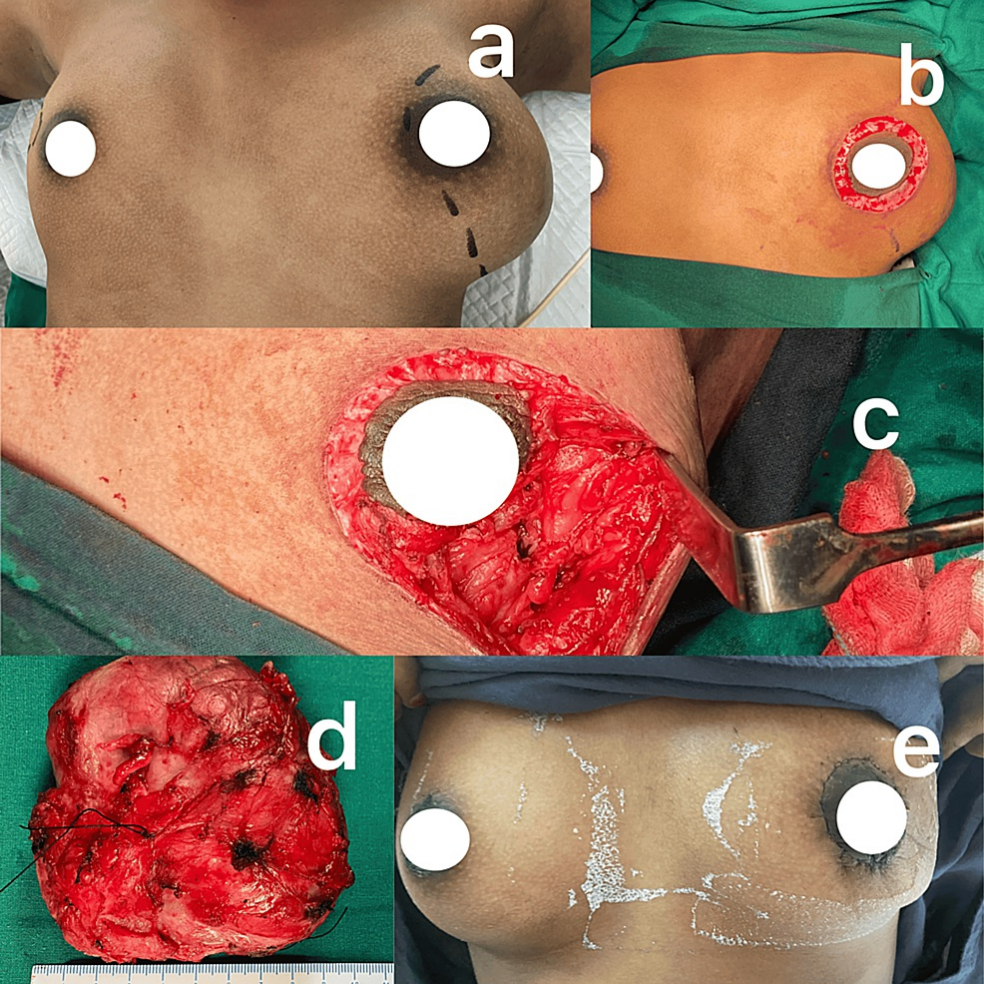
Left breast juvenile fibroadenoma. (a) Preoperative image with tumor outlined in dotted lines, (b) intraoperative incision, (c) cavity after excision, (d) tumor after excision, and (e) 1-week postoperative image.

**Figure 2:**
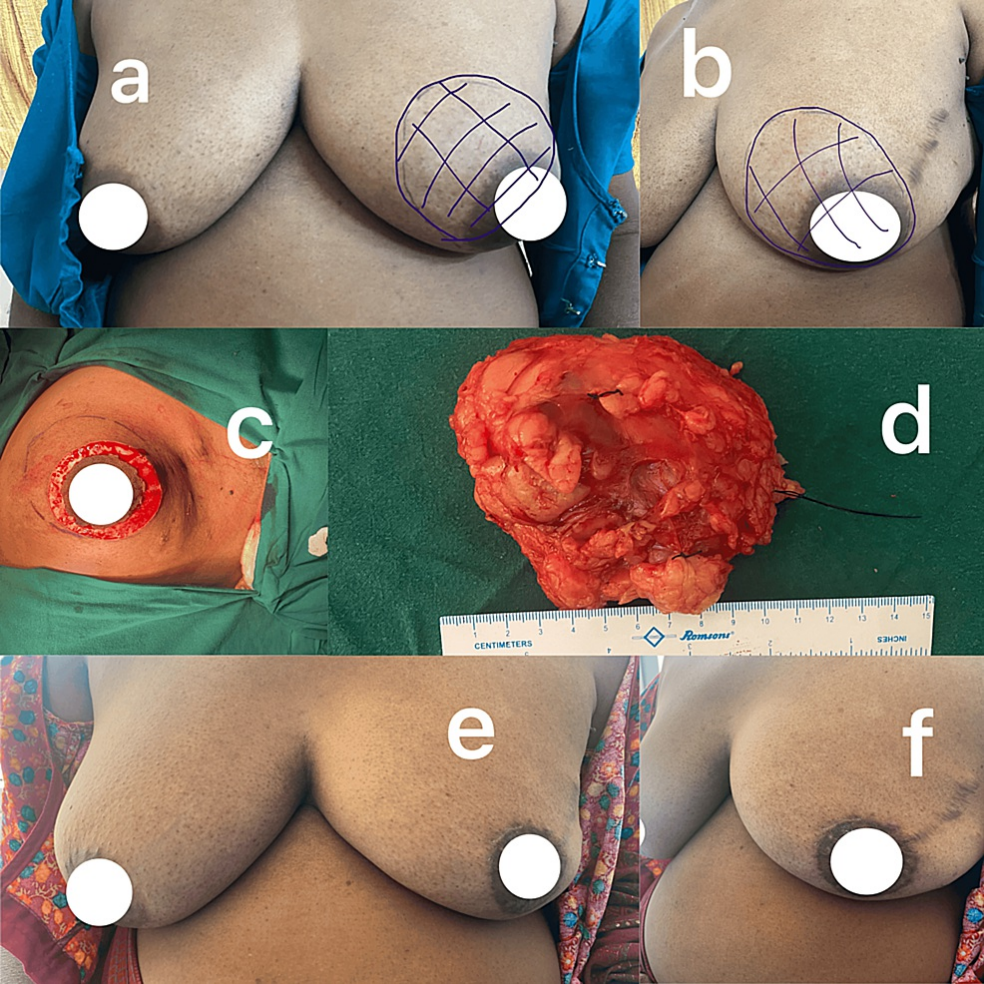
Giant fibroadenoma. (a) Preoperative image, (b) intraoperative circular incision, (c) cavity after excision, (d) tumor after excision, (e) 1-week postoperative image, and (f) 1-month postoperative image.

**Figure 3:**
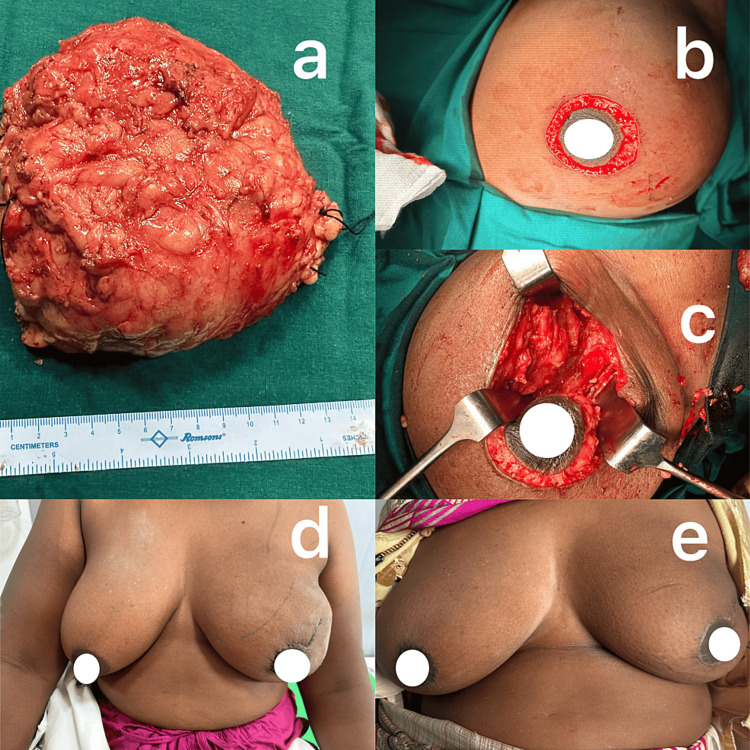
Multicentric fibroadenoma. (a) Preoperative image, (b) intraoperative markings, (c) circular incisions, (d) tumor post-excision, (e) postoperative image (1 week later), and (f) postoperative image (3 months later).

**Figure 4:**
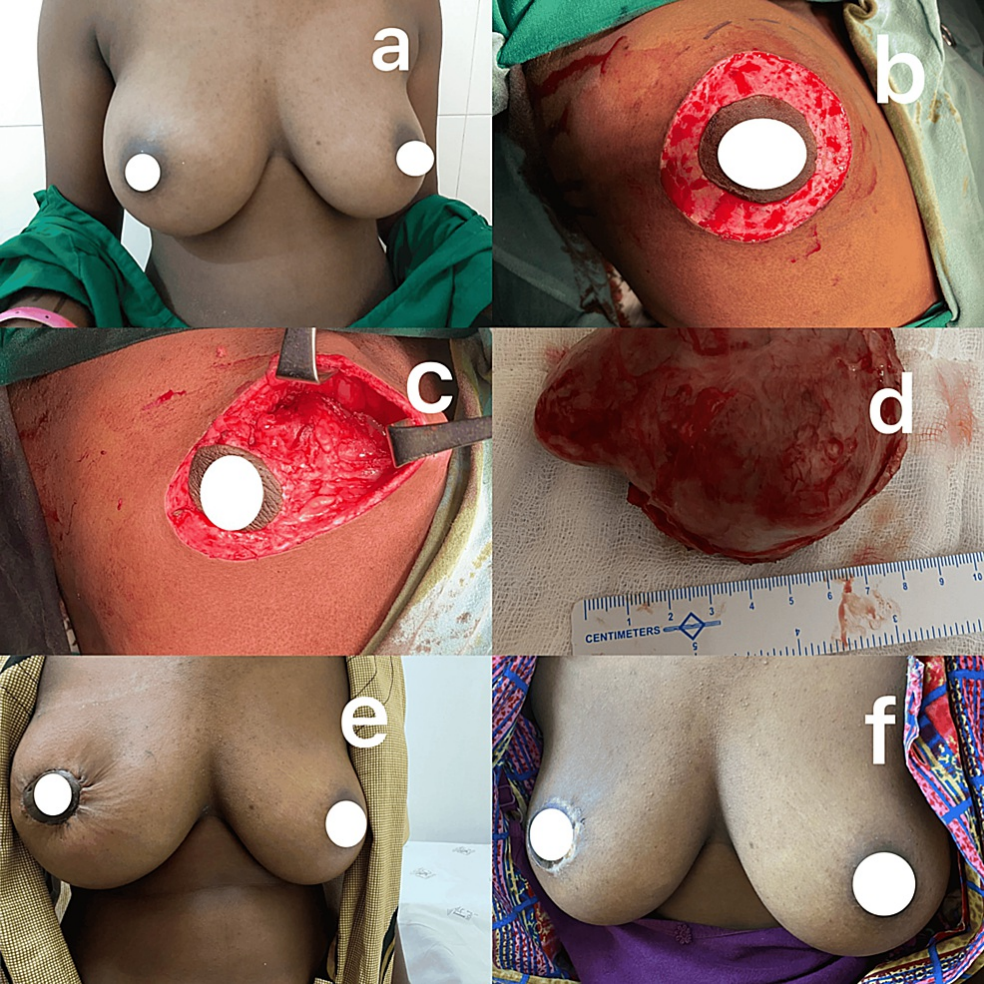
Left breast recurrent phyllodes tumor. (a) Preoperative image in anterior view, (b) preoperative image in lateral view (note the previous radial scar), (c) intraoperative circular incision, (d) tumor with margins marked with silk, (e) postoperative image (anterior view) after 3 months, and (f) postoperative image (lateral view) after 3 months.

The average size of the tumors in the 16 patients was 7.5 cm. Two patients were young adults (Figure 1) with giant fibroadenomas (sizes 10 cm × 7 cm and 10 cm × 9 cm, respectively) in the upper inner quadrant (Figure 2), while two other patients had multicentric tumors (Figure 3). Two patients had recurrent lesions that were initially diagnosed as benign phyllodes tumors by core needle biopsy but were upgraded to borderline phyllodes tumors after wide local excision. Three patients had recurrent phyllodes tumors that were initially operated on by procedures that used radial or circumareolar incisions (Figure 4). All three recurrences were the first recurrence. All margins were free of tumor in seven out of eight phyllodes lesions; the remaining case had one close margin.

**Figure 5:**
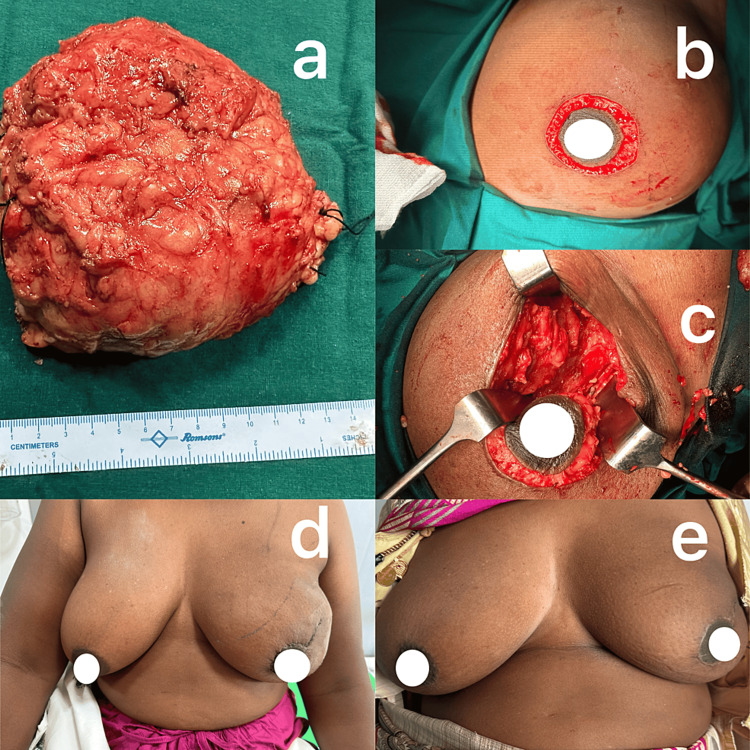
Adenolipoma of left breast. (a) Tumor with margins marked with silk, (b) intraoperative circular incisions, (c) cavity after tumor removal, (d) 1-week postoperative image, and (e) 3-months postoperative.

Three patients had both phyllode tumors and fibroadenoma (Table 1). The round block technique was used for the bigger lesions, while circumareolar incisions were used for the smaller lesions. One patient who was initially diagnosed with a phyllodes tumor was found to have adenolipoma of the breast based on the biopsy report (Figure 5). All margins were free of tumors in seven out of eight phyllode lesions, of which one had a close margin. Radiotherapy was not recommended in any cases due to the good margin status of the phyllodes.

**Table 1:** Distribution of benign breast diseases in our case series.

Type of disease	Preoperative diagnosis	Postoperative diagnosis
Fibroadenoma	5	4
Phyllodes tumor	8	8
Both fibroadenoma and phyllodes tumor	3	3
Adenolipoma	0	1
Total	16	16

Breast symmetry was maintained in all patients, and no nipple areolar necrosis was noted. One patient had partial depigmentation around the nipple-areola complex (Figure 4). Nipple sensation was acceptable in all cases. Cosmetic results were assessed immediately after surgery and during three months of follow-up, and the results were good in all patients. A comparison of the preoperative and postoperative images of patients' breasts showed that symmetry was maintained, and the results were aesthetically acceptable.

## Discussion

Women with benign breast lumps who are undergoing surgery have concerns about postoperative outcomes in terms of recurrence, scarring, and breast asymmetry. Various surgical techniques for the excision of lesions include the use of an inframammary approach, mammoplasty, periareolar incision with extension, and so forth. Oncoplastic techniques have been shown to be useful in achieving margin clearance, obtaining good cosmetic outcomes, and maintaining symmetry with less morbidity.

Additional factors to be considered during management are the complete excision of large tumors, preservation of the nipple-areolar complex, and maintenance of breast symmetry. Avoiding ugly or prominent scars may be desired by all women, especially young women [12]. The round block mammoplasty technique is an oncoplastic procedure that is considered safe for the excision of lesions in all quadrants of the breast while also aiding in aesthetic outcomes and patient satisfaction [13]. Oncoplastic techniques serve a major role in the management of breast cancer surgeries, and these techniques can also be applied in the management of large benign breast lumps.

Phyllodes tumor (serocystic disease of Brodie/cystosarcoma phyllodes) accounts for almost 2-3% of all fibroepithelial diseases. These tumors may be benign, borderline, or malignant, but the majority (60-70%) are benign. Recurrence rates are very high for phyllodes tumors, especially in cases with positive margins and incomplete excision. Benign phyllodes have a recurrence rate of 24-44%, borderline phyllodes tumors have a recurrence rate of 14-25%, and malignant phyllodes tumors recur in 12-54% of cases [14]. Phyllodes tumors tend to have a higher grade after recurrence.

The round block technique provides better access to breast lesions. Consequently, wide local excision of phyllodes tumors is feasible, which allows achieving margin clearance and thus reducing the risk of recurrence. The round block technique stands as a cornerstone therapy in the management of phyllodes tumors. Eleven out of 16 patients in our study were managed by this technique and had no recurrence during the follow-up period. Phyllodes tumor patients should be followed up regularly according to NCCN guidelines, as was done in our study.

Multicentric tumors are defined as tumors present in more than one quadrant of the breast. Excision of all lesions without compromising the margins and symmetry of the breast is essential, but it is difficult to achieve with traditional surgical techniques. Mastectomy is sometimes warranted in these patients. Multicentric lesions are well addressed by the round block technique [15]. In our study, two patients with multicentric tumors were operated on, and the technique enabled the avoidance of mastectomy in these patients.

In a comparative study between reduction mammoplasty and the round block technique [16], the latter procedure showed less morbidity, a lower rate of complications and thus no delay in radiotherapy, and better cosmesis. In addition, contralateral breast surgery for symmetrization was mostly unneeded. In our case series, none of the 16 patients required opposite breast reduction because immediate breast symmetry was achieved by the round block technique alone.

In a previous study with 49 patients, Lim et al. [17] reported that the mean clinical size of tumors was 4.72 cm. We observed an average tumor size of 7.5 cm, which underscores the scope and benefit of the volume-displacing round block technique. Niaz et al. [18] devised a surgical algorithm for large nonmalignant breast tumors, and according to this algorithm, a tumor-to-breast size ratio of up to 70% will allow a good to excellent cosmetic outcome. In cases in which the breast size is too small or almost totally occupied by the tumor, a simple mastectomy followed by immediate reconstruction can be considered. In our study, oncoplastic surgery enabled the avoidance of mastectomy in all patients.

Our study has some limitations. It is a case series of 16 patients and not a comparative study; hence, the results cannot be generalized to a larger population. A prospective study in the future would yield better outcomes. In addition, the duration of our entire study was also a limiting factor that needs to be taken into account.

## Conclusions

The round block technique, or donut lumpectomy, is a useful technique in challenging cases such as giant fibroadenoma, multicentric tumors, and recurrent phyllodes tumors. Although this technique was primarily designed for breast-conserving surgery in breast cancer, it is useful in benign breast diseases, especially in cases with large, recurrent, and multicentric lesions, due to providing good tumor clearance and immediate restoration of breast symmetry. The round block technique should be considered a cornerstone therapy in the management of benign breast lesions such as giant fibroadenoma, multicentric tumors, and recurrent phyllodes tumors. Mastectomy was avoided in all the patients in our case series, eventually decreasing the bad psychological effects and providing an acceptable cosmetic outcome.
